# Atypical Extension to the Chest Wall of Descending Necrotizing Mediastinitis: A Rare Case in a 16-Year-Old Woman

**DOI:** 10.7759/cureus.79559

**Published:** 2025-02-24

**Authors:** Mouad Gourti, Imane Lefqih, FZ Ammor, Mouhssin Makloul, Elmehdi Maidi

**Affiliations:** 1 Surgery Department, Hassan II Medical Center, Medical University of Agadir, Agadir, MAR; 2 Thoracic Surgery Department, Medical University of Agadir, Agadir, MAR

**Keywords:** chest wall invasion, deep neck infection, descending necrotizing mediastinitis, invasive surgical debridement, odontogenic infection

## Abstract

Descending necrotizing mediastinitis (DNM) is a severe and life-threatening infection requiring urgent diagnosis and aggressive management. We present the case of a 16-year-old woman with no prior medical history who developed DNM following an odontogenic infection, complicated by an unusual and rare extension to the chest wall. Such an atypical spread of infection is infrequent and poses additional diagnostic and therapeutic challenges. The use of anti-inflammatory drugs likely masked early symptoms, delaying recognition. Imaging confirmed extensive mediastinal involvement, necessitating prompt surgical intervention. The patient underwent aggressive surgical debridement via cervicothoracic approaches, followed by intensive postoperative care, leading to a favorable outcome. This case highlights the importance of early recognition and a multidisciplinary approach in DNM management, particularly when faced with atypical extensions beyond the mediastinum.

## Introduction

Descending necrotizing mediastinitis (DNM) is a rare but aggressive form of deep neck infection that spreads rapidly along the fascial planes into the mediastinum. It is most commonly associated with odontogenic or oropharyngeal infections and, if left untreated, carries a high mortality, a rate of 20%-40% as described in the literature [1,2]. While DNM typically remains confined to the mediastinum, cases with unusual extensions, such as the invasion of the chest wall, are exceedingly rare. These atypical presentations pose significant diagnostic and therapeutic challenges, often requiring a multimodal approach that includes broad-spectrum antibiotic therapy, aggressive surgical debridement, and intensive care support. There is no standardized surgical management for this condition [1,3].

Here, we describe the case of a young woman with no underlying conditions who developed DNM following a dental abscess, which progressed rapidly despite initial medical treatment. The infection’s unusual extension to the chest wall highlights the need for early imaging, prompt surgical management, and a coordinated multidisciplinary approach to optimize outcomes.

## Case presentation

A 16-year-old woman with no prior medical history presented with a rapidly progressing deep neck infection complicated by descending necrotizing mediastinitis (DNM) and atypical extension to the chest wall. One week before admission, she developed a dental infection, initially treated with anti-inflammatory medications. Over the following days, she experienced progressive swelling at the base of the neck, dysphagia, dyspnea, and fever. Despite antibiotic therapy with amoxicillin-clavulanic acid, symptoms worsened, prompting hospital admission.

**Figure 1:**
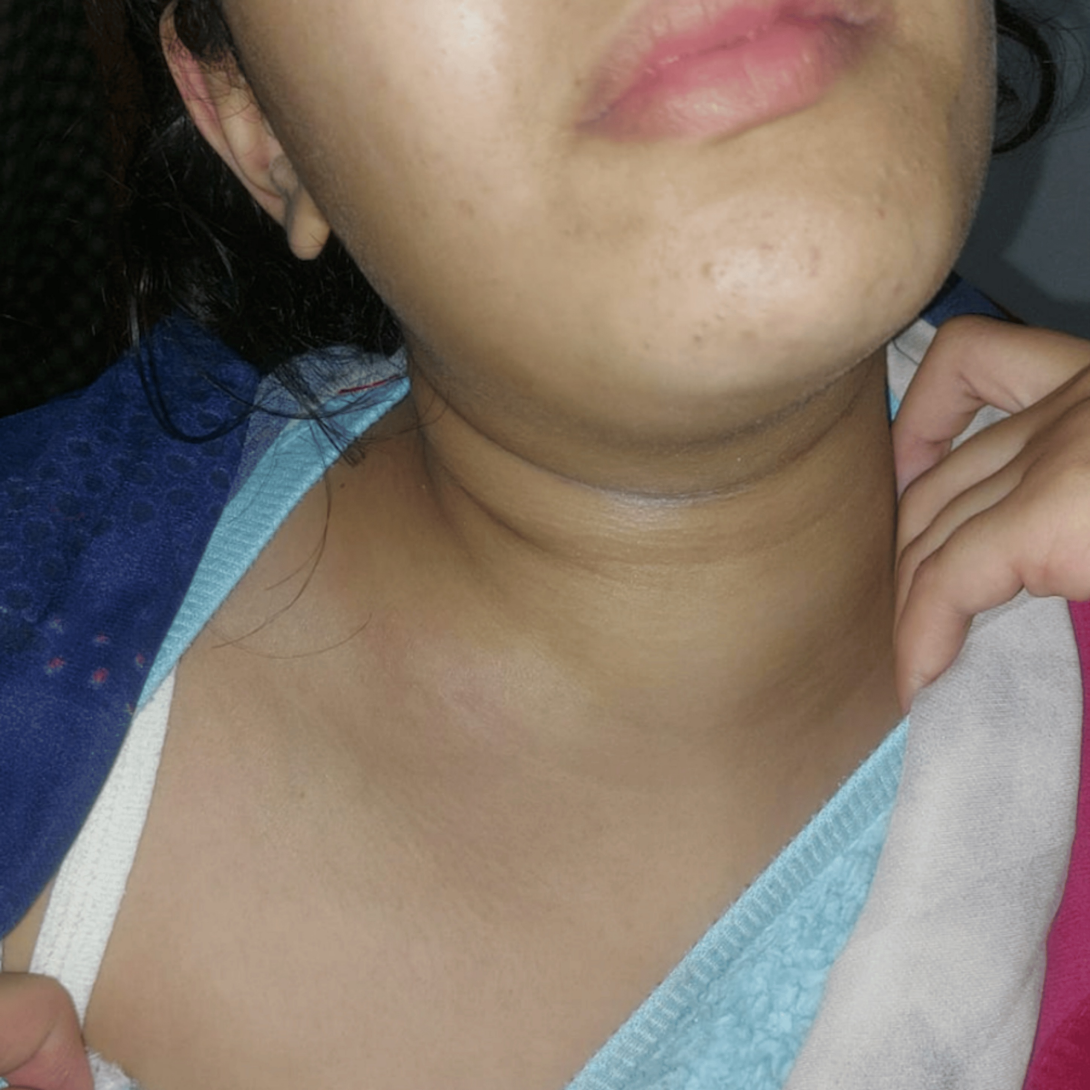
Cervical Swelling With Atypical Axillary Extension in Descending Necrotizing Mediastinitis Clinical image showing cervical swelling with an unusual extension to the axillary region, highlighting the atypical spread of infection in a case of descending necrotizing mediastinitis

On examination, the patient was alert and hemodynamically stable, with a blood pressure of 90/50 mmHg, a heart rate of 95 beats per minute (bpm), a temperature of 37.6°C, and a normal oxygen saturation. A tender, erythematous swelling was noted in the lower right thoracic region, with diffuse subcutaneous emphysema extending over the chest wall (Figure 1).

**Figure 2:**
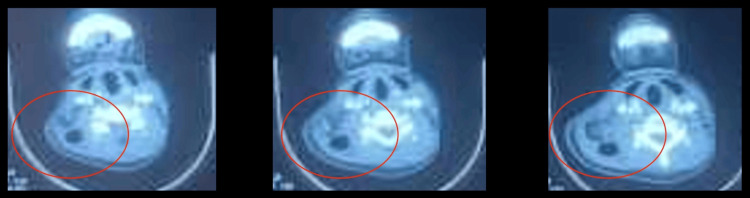
Cervical CT Scan Findings of Descending Necrotizing Mediastinitis in Our Patient Axial and coronal CT images revealing a high lateral cervical collection extending inferiorly, with the presence of air bubbles but without contrast extravasation, indicative of descending necrotizing mediastinitis

**Figure 3:**
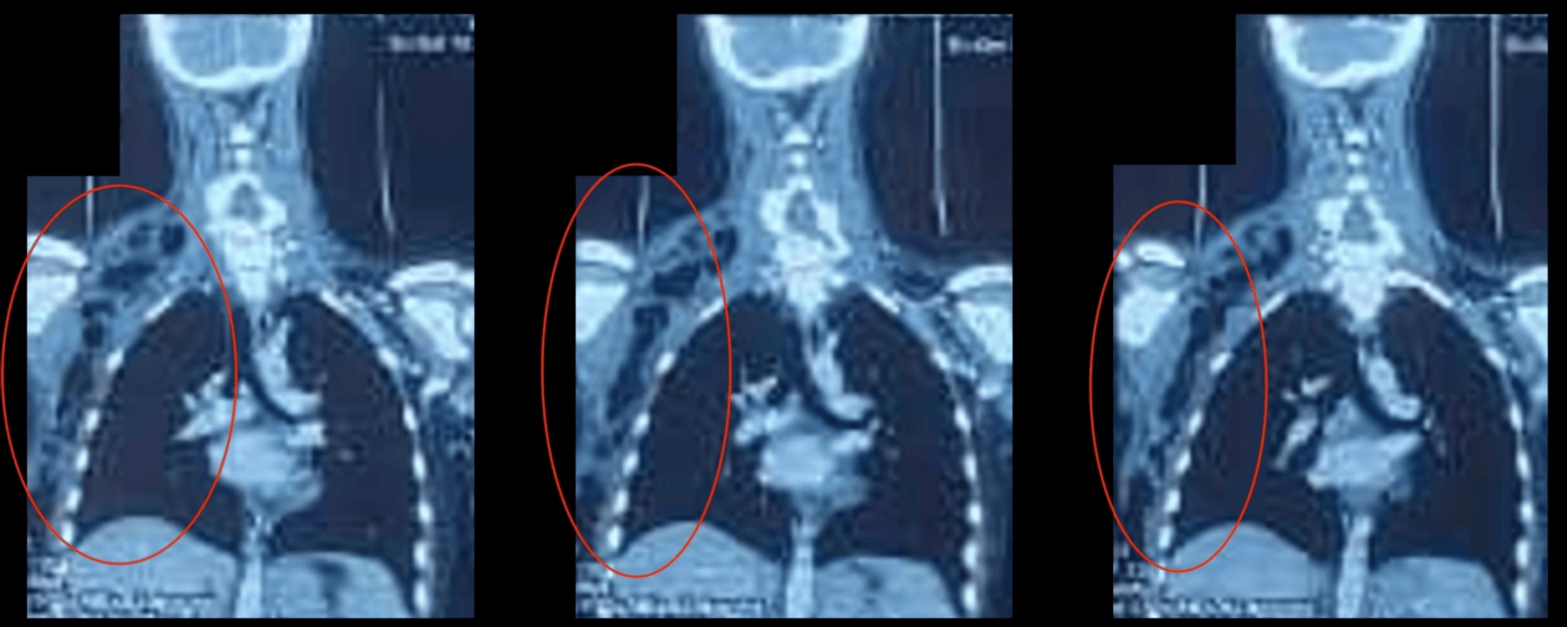
Sagittal Thoracic CT Scan Showing Subcutaneous and Mediastinal Emphysema in Our Patient Sagittal CT scan of the thorax demonstrating subcutaneous and mediastinal emphysema with fluid collections, without the evidence of pleural empyema

Laboratory findings showed leukocytosis (18,000 white blood cells {WBC}/mm³) and an elevated C-reactive protein (CRP, 256 mg/L), with a normal coagulation profile. Cervicothoracic CT imaging revealed necrotizing fasciitis in the cervical region with multiple abscess collections extending to the axillary region, chest wall, and superior mediastinum (Figure 2 and Figure 3).

**Figure 4:**
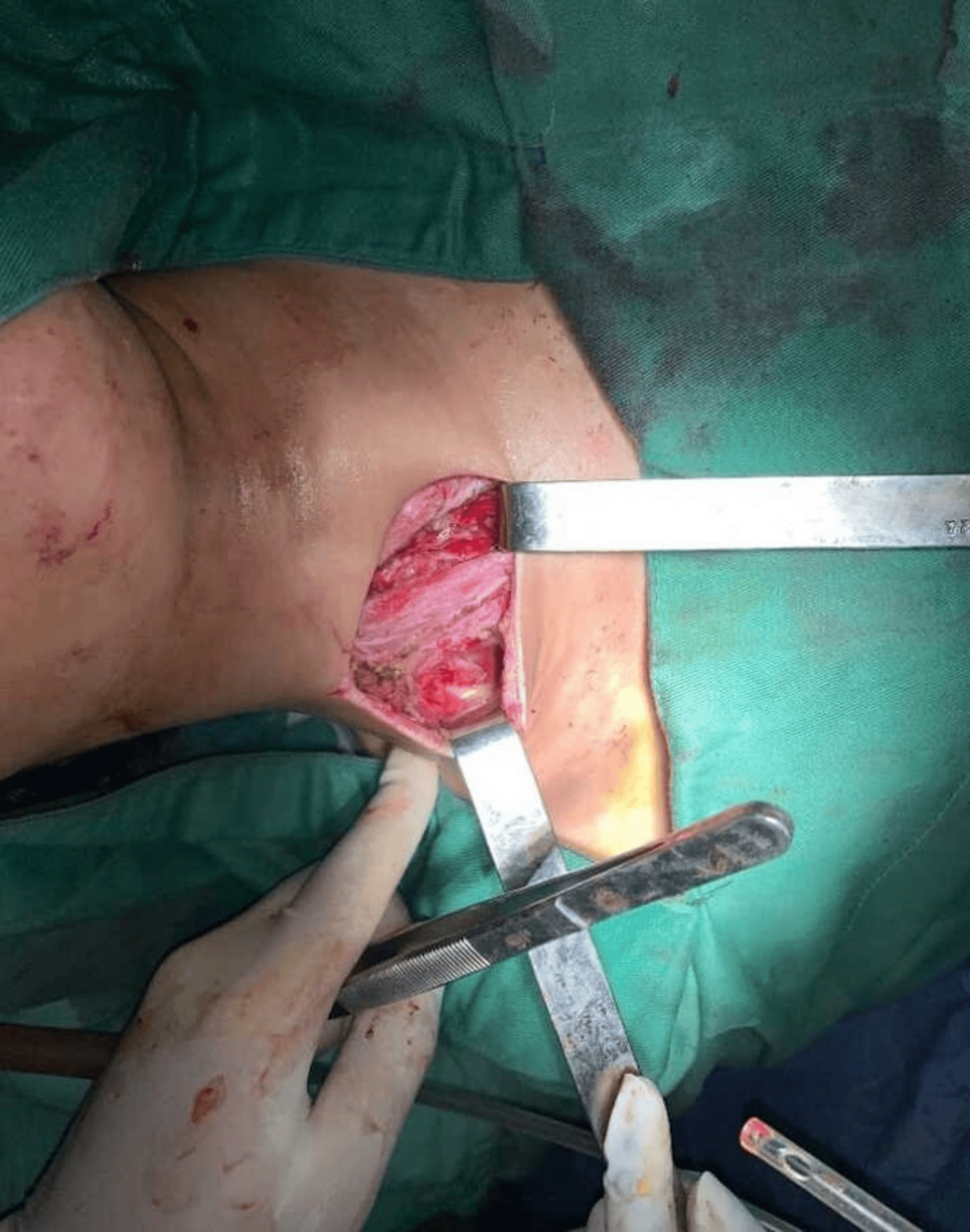
Cervical Incision for Drainage and Superior Mediastinum Exploration Intraoperative image showing a cervical incision performed for the drainage of the cervical collections and the exploration of the superior mediastinum in the management of descending necrotizing mediastinitis

**Figure 5:**
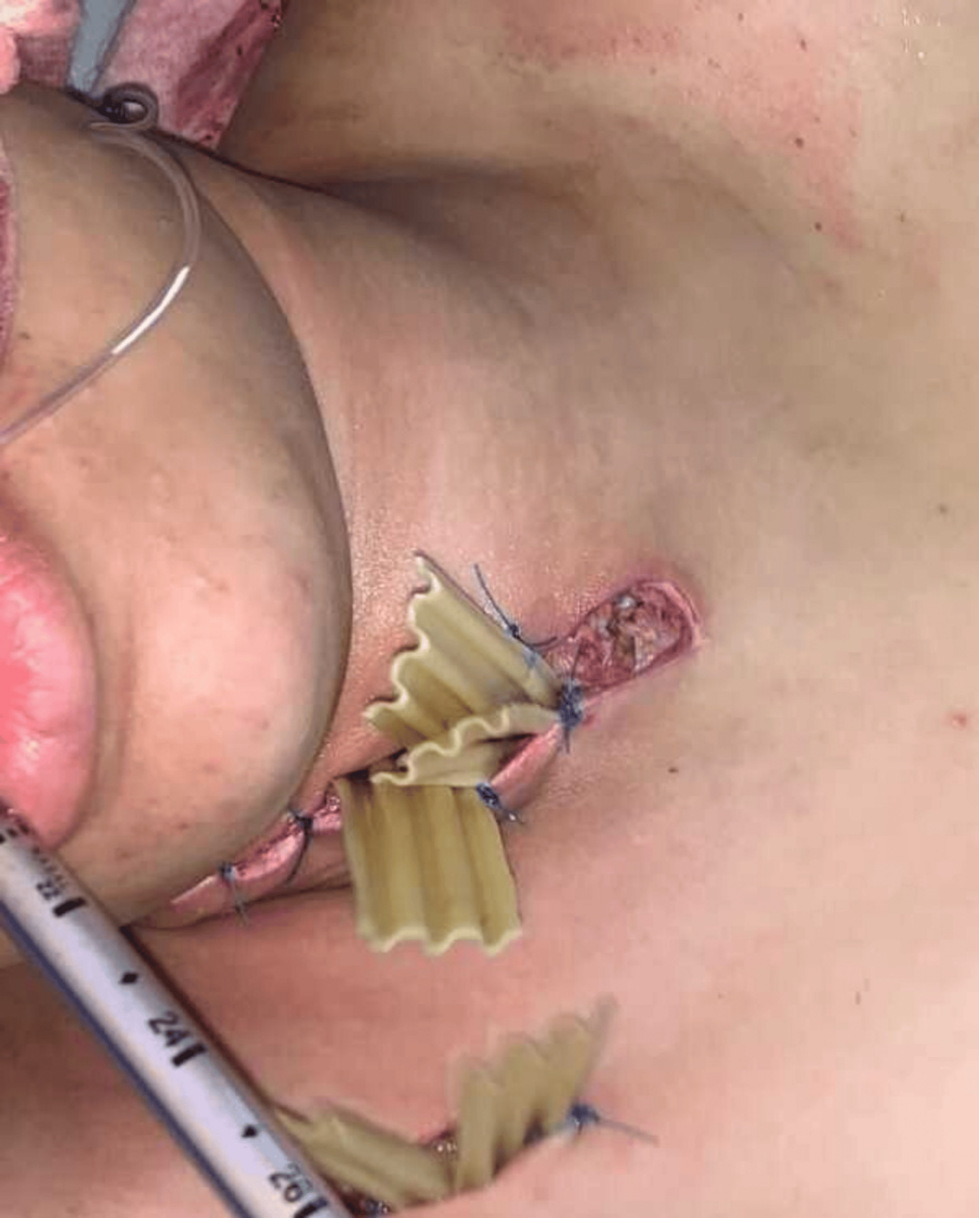
Postoperative View of Delbet Drain With Primary and Counter-Incision Image taken immediately after surgery, before dressing application, showing the Delbet drain in place along with the primary incision and counter-incision used for optimal drainage in the management of descending necrotizing mediastinitis

The patient was urgently taken to the operating room under general anesthesia with orotracheal intubation. Due to intraoperative hypotension (70/40 mmHg), a norepinephrine infusion was initiated. We performed a right lateral cervicotomy with direct mediastinal exploration, ensuring adequate drainage. Given the extent of the infection, additional incisions and counter-incisions were made at the contralateral cervical region, axillary area, and lateral thoracic wall to allow for the optimal evacuation of purulent collections. Surgical exploration revealed extensive purulent collections, necessitating high cervical drainage behind the right sternocleidomastoid muscle, bilateral supraclavicular drainage, and superior mediastinal exploration via a suprasternal incision. Additionally, a right-sided chest wall incision was performed for the debridement and drainage of foul-smelling pus. The procedure included extensive irrigation with saline, betadine, and hydrogen peroxide and the placement of seven Delbet drains for postoperative management (Figure 4 and Figure 5).

Postoperatively, the patient was admitted to the intensive care unit, where she was initially intubated, ventilated, and sedated. Extubation was performed successfully at 24 hours without respiratory distress. Empiric intravenous (IV) antibiotic therapy with ceftriaxone (2 g), gentamicin, and metronidazole was initiated. Supportive care included hyperproteic enteral nutrition, intensive wound care three times daily, and motor and respiratory physiotherapy. Serial laboratory monitoring showed a progressive decline in CRP levels (170 mg/L on day 3, 90 mg/L on day 7, and 30 mg/L on day 14), with the normalization of the leukocyte count (Table 1).

**Table 1:** Laboratory Evolution of Our Patient Managed for Descending Necrotizing Mediastinitis With Chest Wall Extension This table presents the laboratory evolution of a patient diagnosed with descending necrotizing mediastinitis with chest wall extension, managed with surgical debridement and broad-spectrum antibiotics. The white blood cell (WBC) count initially showed leukocytosis, progressively decreasing with treatment. C-reactive protein (CRP) levels, a marker of inflammation, followed a downward trend over two weeks, reflecting clinical improvement

Day	White Blood Cells (WBC/mm³)	C-reactive Protein (CRP, mg/L)	Hemoglobin (g/dL)
0	18,000.0	256.0	9.0
1	19,000.0	-	-
3	-	170.0	-
7	13,000.0	90.0	11.0
14	9,000.0	30.0	-
Normal range	4,000-11,000 WBC/mm³	<10 mg/L	Male: 13.8-17.2 g/dL; female: 12.1-15.1 g/dL

Microbiological search and culture results for our patient did not reveal a specific causative germ nor tuberculosis and actinomycosis (Table 2).

**Table 2:** Bacteriological Test Results of Our Patient Bacteriological test performed on a patient with acute descending necrotizing mediastinitis. The test aims to identify the causative pathogens and guide targeted antibiotic therapy. In this case, the test results were negative MGG: May-Grünwald-Giemsa

Category	Result
Nature of the sample	Pus
Appearance	Hematic
Direct examination: leukocytes	Numerous
Direct examination: red blood cells	Numerous
Direct examination: epithelial cells	Rare
Direct examination: yeasts	Absent
Examination after MGG staining: neutrophil polymorphonuclear cells	80%
Examination after MGG staining: lymphocytes	20%
Gram and methylene blue staining	No pathogenic germs detected
Culture on standard media	No pathogenic germs detected
Culture on anaerobic incubated media	No anaerobic germs detected
Conclusion: the presence of pathogenic germs	No specific pathogenic germs isolated
Conclusion: neutrophil polymorphonuclear cell predominance	90%
Conclusion: recommendation	To be correlated with clinical and therapeutic data
Antibiogram	-

**Figure 6:**
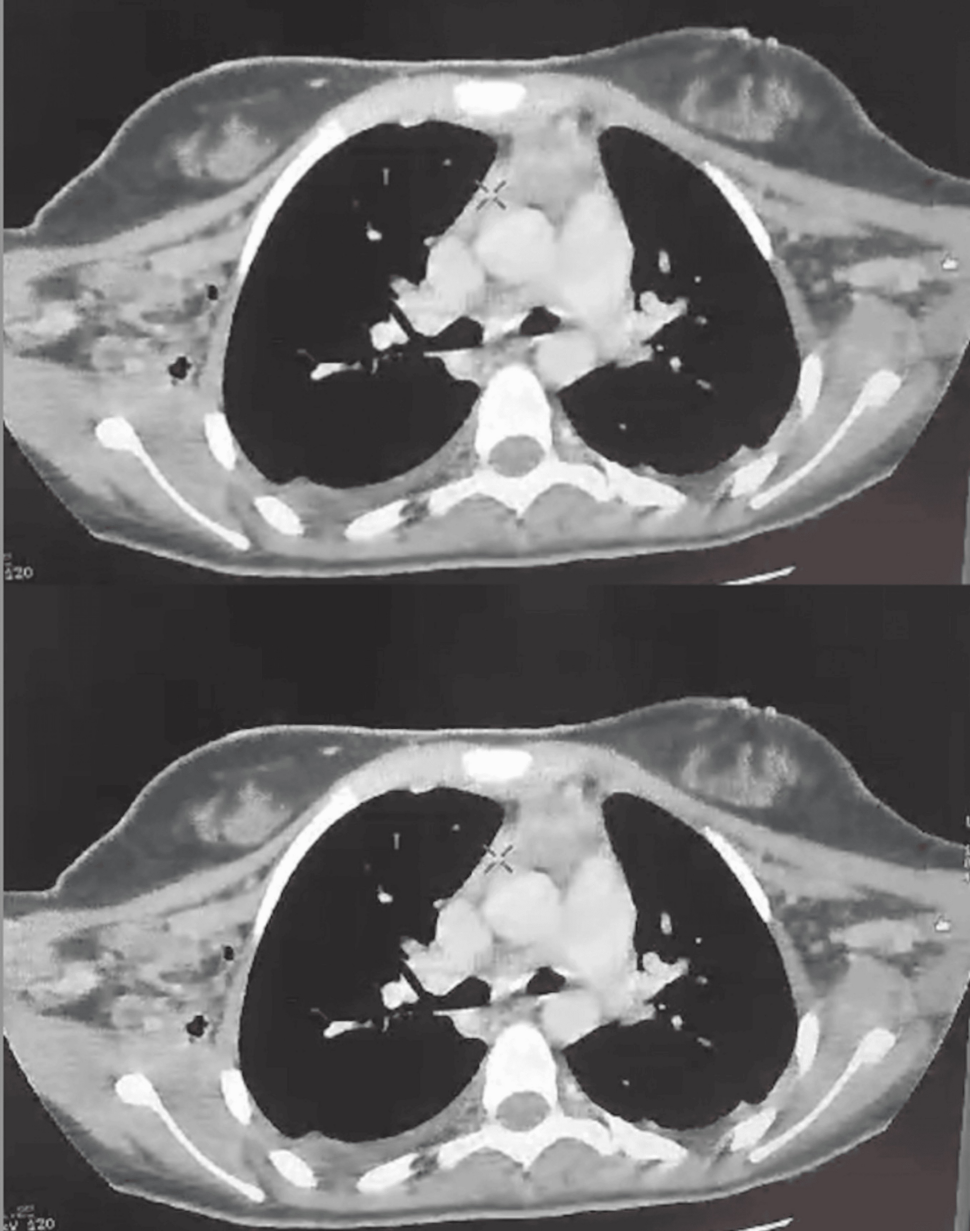
Postoperative CT Scan of Our Patient With Descending Necrotizing Mediastinitis (Day 3) This postoperative CT scan, performed on day 3 after surgery, showed the absence of new fluid collections or significant mediastinal widening, suggesting a favorable postoperative evolution

A postoperative day 3 CT scan revealed no signs of residual mediastinal collection or new complications, suggesting a favorable early response to surgical management; continuous monitoring remains essential to ensure optimal recovery (Figure 6).

The patient was transferred to the general surgery ward for continued care and was discharged on postoperative day 14 in stable condition with no complications. In our case, antibiotic therapy was administered for a total duration of six weeks following a stepwise approach: intravenous (IV) antibiotics for the first three weeks to ensure broad-spectrum coverage against aerobic and anaerobic pathogens commonly involved in descending necrotizing mediastinitis. This case highlights the importance of early recognition, aggressive surgical debridement, and a multidisciplinary approach in the management of descending necrotizing mediastinitis with atypical chest wall involvement.

## Discussion

Descending necrotizing mediastinitis (DNM) is a rapidly progressing, life-threatening infection resulting from an oropharyngeal or odontogenic source, spreading through the deep cervical fascial planes into the mediastinum [1,2]. The continuity of these fascial structures allows for the rapid dissemination of the infection, often leading to severe septic complications, particularly when diagnosis and intervention are delayed [2,3]. Despite advancements in intensive care, imaging modalities, and surgical techniques, DNM continues to be associated with significant morbidity and mortality [1,4].

While DNM is classically confined to the mediastinum, rare cases demonstrate an extension to the thoracic wall, an atypical and aggressive presentation [3,4]. The presence of subcutaneous emphysema in such cases suggests that both air and infection have been dissected through the fascial planes, reaching the parietal thoracic region [5,6]. This unusual dissemination pathway highlights the virulence of the infection and the potential for extensive tissue destruction, necessitating urgent surgical intervention.

The cornerstone of DNM management remains early surgical drainage and debridement. Cross-sectional imaging, particularly contrast-enhanced CT scans, plays a critical role in evaluating the extent of infection and guiding the surgical approach [6,7]. Standard treatment includes aggressive surgical debridement, broad-spectrum antibiotic therapy targeting polymicrobial infections, and intensive postoperative wound care [8].

A multidisciplinary approach is essential for optimizing patient outcomes. This includes collaboration between thoracic surgeons, intensivists, infectious disease specialists, and radiologists [1,6]. In cases with thoracic wall involvement, extended surgical techniques, including fasciotomy or muscle flap reconstruction, may be required to manage extensive soft tissue necrosis [6,7].

This case underscores the importance of early recognition and intervention in DNM, particularly when the infection exhibits atypical dissemination. The rare extension to the thoracic wall necessitates a heightened clinical suspicion and a tailored surgical approach. Integrating prompt diagnosis, aggressive surgical management, and comprehensive supportive care is crucial for improving survival in patients with this highly lethal condition [8,9].

## Conclusions

This case underscores the critical importance of the early recognition and aggressive management of descending necrotizing mediastinitis, particularly in rare presentations involving chest wall extension. A multidisciplinary approach, integrating surgical intervention, intensive care support, and physiotherapy, plays a vital role in optimizing patient outcomes. The polymicrobial nature of DNM highlights the importance of broad-spectrum antibiotic coverage in initial management, especially in cases where prior antimicrobial exposure may obscure microbiological identification. Despite the severity of the infection, timely diagnosis and prompt surgical debridement resulted in a favorable recovery, highlighting the necessity of rapid and coordinated treatment strategies in managing this life-threatening condition.
